# Genome Sequences of Four *Staphylococcus capitis* NRCS-A Isolates from Geographically Distant Neonatal Intensive Care Units

**DOI:** 10.1128/genomeA.00501-15

**Published:** 2015-08-06

**Authors:** H. Lemriss, S. Lemriss, P. Martins-Simoes, M. Butin, L. Lahlou, J.-P. Rasigade, A. Kearns, O. Denis, M. Deighton, A. Ibrahimi, F. Laurent, S. El Kabbaj

**Affiliations:** a Biotechnology Laboratory (Medbiotech), Medical and Pharmacy School, University Mohammed V de Rabat, Rabat, Morocco; b Department of Biosecurity PCL3, Laboratory of Research and Medical Analysis of the Fraternal of Gendarmerie Royale, Rabat, Morocco; c International Centre for Research in Infectious diseases, INSERM, University of Lyon, Lyon, France; d Department of Clinical Microbiology, Northern Hospital Group, Hospices Civils de Lyon, Lyon, France; e National Reference Center for Staphylococci, Hospices Civils de Lyon, Lyon, France; f Public Health England, Colindale, London, United Kingdom; g Erasme Hospital, Université Libre de Bruxelles, Brussels, Belgium; h RMIT University, Bundoora, Victoria, Australia

## Abstract

*Staphylococcus capitis* pulsotype NRCS-A was previously reported as a frequent cause of late-onset sepsis in neonatal intensive care units (NICUs) worldwide. Here, we report the whole-genome shotgun sequences of four *S. capitis* pulsotype NCRS-A strains, CR03, CR04, CR05, and CR09, isolated from Belgium, Australia, the United Kingdom, and France, respectively.

## GENOME ANNOUNCEMENT

Coagulase-negative staphylococci (CoNS) are the most frequently encountered pathogens in neonatal intensive care units (NICUs) (1). However, in the NICU setting, recent studies have indicated that methicillin-resistant *Staphylococcus capitis* might emerge as a significant pathogen, causing late-onset sepsis (LOS) in several neonatal intensive care units in France, the Netherlands, and Australia (2–4).

A study in French NICUs demonstrated the spread of a single clonal population of methicillin-resistant *S. capitis* (pulsotype NRCS-A) associated with reduced susceptibility to vancomycin, which is the first line of antibiotics used in cases of LOS. Moreover, this clone has also been recently identified in NICUs in Belgium, the United Kingdom, and Australia, suggesting a worldwide distribution (5, 6).

In this report, we present the draft genome sequences of four *S. capitis* (pulsotype NRCS-A) strains (CR03, CR04, CR05, and CR09) isolated from blood cultures from four neonates hospitalized in NICUs in Belgium, Australia, the United Kingdom, and France, respectively.

All *S. capitis* strains were grown in blood agar at 37°C, and genomic DNA was extracted using the PureLink genomic DNA kit (Invitrogen), according to the manufacturer’s recommended protocol. The quantity of DNA was determined using a NanoVue Plus (HVD Lifesciences), and 1 µg of DNA was used to sequence the whole genome of each strain. The 454-shotgun libraries were prepared from the extracted genomic DNA following GS rapid library protocol (Roche 454; Roche).

The genome sequence of each *S. capitis* strain was determined by high-throughput sequencing performed on a Genome Sequencer FLX+ system (454 Life Sciences/Roche) using FLX Titanium reagents, according to the manufacturer’s protocols and instructions. *De novo* assemblies were performed using the Roche Newbler (version 2.9) software package, and the sequencing results are summarized in Table 1.

**TABLE 1:** Summary of genome sequencing results in the present study

*S. capitis* strain	Country source	Reads (Mb)	Fold coverage (×)	No. of contigs	Genome size (bp)	G+C content (%)	Accession no.
CR03	Belgium	141,728	30	31	2,508,352	32.81	CTEB01000000
CR04	Australia	132,280	30	38	2,512,289	32.80	CTEM01000000
CR05	United Kingdom	139,569	31	39	2,543,917	32.84	CTEO01000000
CR09	France	132,205	30	34	2,490,458	32.82	CTEL01000000

An automatic syntactic and functional annotation of the draft genome was performed using the MicroScope platform pipeline (7, 8). The syntactic analysis combines a set of programs, including AMIGene (9), tRNAscan-SE (10), RNAmmer (11), Rfam scan (12), and Prodigal software (13) to predict genomic objects that are mainly coding sequences (CDSs) and RNA genes. More than 20 bioinformatics methods were used for functional and relational analyses. The homology search was performed in the generalist databank UniProt (14) and in more specialized databases, such as COG (15), InterPro (16), PRIAM profiles for enzymatic classification (17), prediction of protein localization using TMHMM (18), SignalP (19), and PSORTb (20) tools.

The chromosome of strain CR03 (ENA accession no. CTEB01000000) contains 2,575 genes, 2,466 coding sequences (CDSs), 4 rRNAs, and 61 tRNAs; the chromosome of strain CR04 (accession no. CTEM01000000) contains 2,566 genes, 2,457 CDSs, 4 rRNAs, and 60 tRNAs; the chromosome of strain CR05 (accession no. CTEO01000000) contains 2,624 genes, 2,508 CDSs, 4 rRNAs, and 60 tRNAs; and the chromosome of strain CR09 (accession no. CTEL01000000) contains 2,540 genes, 2,432 CDSs, 4 rRNAs, and 59 tRNAs.

### Nucleotide sequence accession numbers.

This whole-genome shotgun project has been deposited at the ENA database under the accession numbers listed in Table 1. The versions described in this paper are in the first versions, under BioProject designation no. PRJEB8618.
